# The associations between low abundance of *Mycoplasma hominis* and female fecundability: a pregnancy-planning cohort study

**DOI:** 10.1186/s12866-022-02545-7

**Published:** 2022-05-05

**Authors:** Xiang Hong, Jiechen Yin, Wei Wang, Fanqi Zhao, Xiaoling Ding, Hong Yu, Xuening Zhang, Bei Wang

**Affiliations:** 1 Department of Epidemiology and Health Statistics, School of Public Health, Southeast University, Key Laboratory of Environmental Medicine and Engineering of Ministry of Education, 87#, Dingjiaqiao Road, Gulou District, Nanjing City, Jiangsu Province China; 2 Maternal and Child Health Center of Gulou District, Nanjing, China; 3 Department of Obstetrics and Gynecology, Zhong Da Hospital, Southeast University, Nanjing, China; 4 Jiangsu Health Development Research Center, Nanjing, Jiangsu China; 5 NHC Key Laboratory of Contraceptives Adverse Reaction Surveillance, 277#, Fenghuangxi Road, Gulou District, Nanjing City, Jiangsu Province China

**Keywords:** *Mycoplasma hominis*, Vaginal microbiome, Fecundability, Time to pregnancy

## Abstract

**Objective:**

To explore the impact of pre-pregnancy vaginal *Mycoplasma hominis* (*M. hominis*) colonization of low abundance on female fecundability.

**Methods:**

In total, 89 females participating in a pre-pregnancy health examination program were included, and their pregnancy outcomes were followed up for 1 year. Vaginal swabs were collected, 16S rRNA genes were sequenced, and *M. hominis* colonization was confirmed by qPCR. Cox models were used to estimate the fecundability odds ratio (FOR) for women with *M. hominis*.

**Results:**

The prevalence of *M. hominis* was 22.47% (20/89), and the abundance was relatively low (the cycle thresholds of the qPCR were all more than 25). In terms of the vaginal microbiome, the Simpson index of the positive group was significantly lower than that of the negative group (*P* = 0.003), which means that the microbiome diversity appeared to increase with *M. hominis* positivity. The relative abundance of *M. hominis* was negatively correlated with *Lactobacillus crispatus* (rho = − 0.24, *P* = 0.024), but positively correlated with *Gardnerella vaginalis*, *Atopobium vaginae* and *Prevotella bivia* (*P* all < 0.05). The cumulative one-year pregnancy rate for the *M. hominis* positive group was lower than that in the negative group (58.96% vs 66.76%, log-rank test: *P* = 0.029). After controlling for potential confounders, the risk of pregnancy in the *M. hominis* positive group was reduced by 38% when compared with the positive group (FOR = 0.62, 95% CI: 0.42–0.93).

**Conclusion:**

The vaginal colonization of *M. hominis* at a low level in pre-pregnant women is negatively correlated with female fecundability.

**Supplementary Information:**

The online version contains supplementary material available at 10.1186/s12866-022-02545-7.

## Introduction

The reduction of female fertility is a complex problem of public health; its potential causes include social, environmental and physiological factors [1, 2]. A meta-analysis showed that the female infertility was associated with vaginal microbiota, [3] but most evidence was derived from case–control studies, which limited the causal inference. Recently, two prospective cohort studies illustrated that the female time-to-pregnancy (TTP) index was associated with the vaginal microenvironment or bacterial vaginitis (BV) status [4, 5]. TTP is a typical indicators reflecting couples’ fecundability, which is defined as a couple’s probability of conception in one menstrual cycle, given regular intercourse and no method of contraception [6]. However, the structure of the vaginal mocribiome is complex, there are hundreds of different species in this environment even among healthy women, and which of them are vital factors with an impact on female fecundability is unknown.

*Mycoplasma* are prevalent, and are the smallest genital *Mollicutes* in the vaginal microbiome; they are unique genera with no cell wall around the cell membrane [7]. *Mycoplasma hominis (M. hominis)* is the most common *Mycoplasma* species in the vagina. Some studies have found that the colonization rate of *M. hominis* is 3.1–15% [8]. Although some evidence suggests that vaginal *M. hominis* infection is associated with BV [9] and preterm birth [10], it is still regarded as an opportunistic pathogen [8] because many asymptomatic females are positive. Our previous preliminary study showed that the colonization of *M. hominis* was associated with the structure of the vaginal microbiota, [11] which suggested that *M. hominis* might play an important role in female health. Inconsistent findings suggest that vaginal *M. hominis* infection is a potential reason for female infertility, [12] but no studies have shown whether vaginal *M. hominis* might affect the female TTP. Therefore we used the data from a pregnancy-planning cohort to explore, among asymptomatic pregnancy-planning females, whether a low abundance of *M. hominis* colonization can impact the TTP, in order to provide an innovative perspective for female infertility prevention.

## Materials and methods

### Study population

A pregnancy-planning cohort study was conducted at the Maternal and Child Health Center of Gulou district, Nanjing, China, from September 2018 to June 2019. All the women who participated to the free pre-pregnancy health examination program and stated that they were ready to attempt pregnancy were included. This program was supported by Chinese government from 2010, which aimed to reduce birth defects rate among population. The original design, organisation and implementation of the program have been described previously [13]. With this program, we set the following exclusion criteria to carry out this study: (1) females who had been pregnant when participating in the program; (2) those who were diagnosed with reproductive malformation, or their male partners with testicular loss; (3) women who were infected with *Treponema pallidum*, cytomegalovirus or *Toxoplasma gondii*; (4) those who did not collect the vaginal swabs because of a menstrual period; (5) women who were diagnosed with BV or colpomycosis, which required medical treatment. A total of 102 women met the criteria and were recruited, after giving informed consent. The Ethics Committee of Zhongda Hospital approved this study (2018ZDSYLL072-P01), all methods were performed in accordance with the declarations of Helsinki. In the follow-up phase, 13 females who gave up attempting pregnancy within 3 months and refused to tell us the exact time they had have kept the desire of pregnancy were excluded. Thus, 89 females were analyzed in this study.

### Vaginal bacterial nucleic acid extraction

Gynecologists used two sterile swabs and a speculum to collect vaginal secretions. The swabs were transferred to a − 80 °C refrigerator, and saved for bacterial nucleic acid extraction. Following the standard procedures in our laboratory, [14] swabs were eluted in 2 ml PBS, and TIANamp Bacteria DNA Kits (Tiangen Biochemical Technology, Beijing, China) were used to extract and purify DNA. The concentrations of DNA were measured through Nanodrop one (Thermo Scientific Co.,Ltd., MA, USA). All the nucleic acid samples were taken successfully, the average concentration is 63.46 ± 10.26 ng/ul.

### 16S rRNA gene sequencing and *M. hominis* identification

The V3–V4 hypervariable regions of the DNA samples were amplified by polymerase chain reaction (PCR) using universal primers (338F: 5′-ACTCCTACGGGAGGCAGC3’ and 806R: 5′-GGACTACHVGGGTWTCTAAT-3′). The PCR products were sequenced on an IlluminaHiseq 2500 platform (Beijing Biomarker Technologies Co. Ltd., Beijing, China). Sequencing data was processed on the Biomarker biocloud platform (www.biocloud.org). Paired-end reads were merged by FLASH (v1.2.7, http://ccb.jhu.edu/software/FLASH/). Some low quality tags were removed using Trimmomatic (v0.33). Denoised sequences were clustered using USEARCH (version 10.0), and tags with similarity ≥97% were regarded as operational taxonomic units (OTUs). Subsequently, the NCBI database was used to annotate taxonomic information with QIIME. The information on OTU relative abundance was normalized using a standard sequence number corresponding to the sample with the fewest sequences. The samples with representative sequences of *M. hominis* were judged as the positive group, and the qPCR method was used to confirm the abundance by cycle threshold value (CT). Considering that the study participants were all asymptomatic, CT < 35 was regarded as positive. The primers (5′-TCACTAAACCGGGTATTTTCTAACAA-3′ and 5′-TTGGCATATATTGCGATAGTGCTT-3′) were used. The ChamQ SYBR qPCR Master Mix (Vazyme Biotechnology Co.,Ltd., Nanjing, China) was used for reaction preparation (20 μl), and a standard amplification program was set up in the Real-Time PCR System (StepOne plus, Applied Biosystems Co., Ltd., USA). There were 3 replicates for each sample, at least two consistent CT values were considered credible results, otherwise, we would repeat the detection.

### Outcome assessment

All the participants were followed up by telephone every 3 months until 1 year later; whether they were pregnant and their last menstrual periods (LMP) were recorded. The TTP was calculated as the interval between the date of participation in the study and the date of the LMP obtained at follow-up (if pregnant within 1 year) or last follow-up date (if not pregnant).

### Statistical analysis

The continuous variables were described as mean ± standard deviation (normal distribution), or median with interquartile range (non-normal distribution); comparison among groups was assessed by t test (normal distribution) or Kruskal–Wallis test (non-normal distribution). The chi-square test was used to test differences in frequency distribution among groups. Alpha diversity indices, including Simpson and Chao1 indices, were calculated by mothur software (version 1.30). A higher Chao1 index and lower Simpson index mean a more diversity for microbiome, the details are shown in the Supplementary text. The binary Jaccard distance, one of the β-diversity indices that focuses on differences in taxonomic abundance profiles from multiple samples, was assessed with QIIME software, and following principal coordinate analyses (PCoAs), and permutational multivariate analysis of variance (PERMANOVA) were used to compare the differences between groups. PCoA is a non-constrained data dimension reduction analysis method, which is also a visualization method to study the similarity or difference of data. Differences between individuals or groups can be observed through PCoA plot, thus it was widely used in microbiome data analysis [15]. The correlations between the abundance of *M. hominis* and other species were evaluated using the Spearman correlation coefficient. Linear discriminant analysis effect sizes (Lefse) were calculated to determine biomarkers between the groups. Kaplan–Meier plots and the log-rank test were used to present the cumulative pregnancy rate among different groups. In order to adjust for some potential confounders, Cox models were used to estimate the fecundability odds ratio (FOR) and the 95% confidence interval (CI). The FOR estimated the odds of becoming pregnant in the current month for participants with or without *M. hominis* infection, conditional on not being pregnant in the previous month, thus FOR < 1 indicated a reduction in fecundability. Model A was adjusted by age, pregnancy history and menstruation regularity and couples’ age differences; Model B was additionally adjusted by vaginal cleanness grading and the Shannon and Simpson index to control for the impact of overall vaginal microenvironment. A two-sided *P* value ≤0.05 was deemed statistically significant.

## Results

### Participants and basic characteristics

Among the 89 women, the average age was 28.60 years. After qPCR confirmation, the *M. hominis* positive rate was 22.47% (20/89). Among these samples, all the CT values were more than 25 (28.32 ± 2.31). The woman’s age, age differences within couples, pregnancy history and menstruation situation were comparable between the *M. hominis* positive and negative groups (*P* > 0.05, Table 1). Although the proportion of vaginal cleanness grades III–IV in positive group was higher than in the negative group, the difference did not have statistical significance (35.0% vs 26.1%, *P* = 0.484).

**Table 1:** The basic characteristics of the participants based on *M. hominis* status

	*M.hominis* negative *N =* 69	*M.hominis* positive *N =* 20	*t/χ2*	*P*
Age, mean ± SD	28.65 ± 3.30	28.45 ± 2.97	0.26	0.800
Age difference	1.02 (− 0.40 ~ 3.41)	1.45 (0.09 ~ 3.20)	0.08^a^	0.963
Pregnancy history, n (%)				
No	62 (91.2)	17 (85.0)	0.15	0.703
Yes	6 (8.8)	3 (15.0)		
Regular menstruation, n (%)			0.49	0.485
No	13 (19.4)	6 (30.0)		
Yes	54 (80.6)	14 (70.0)		
Vaginal cleanness grading, n (%)			0.49	0.484
I-II	51 (73.9)	13 (65.0)		
III-IV	18 (26.1)	7 (35.0)		

^a^ Kruskal–Wallis test

### Vaginal microbiome and *M. hominis* colonization status

**Fig. 1:**
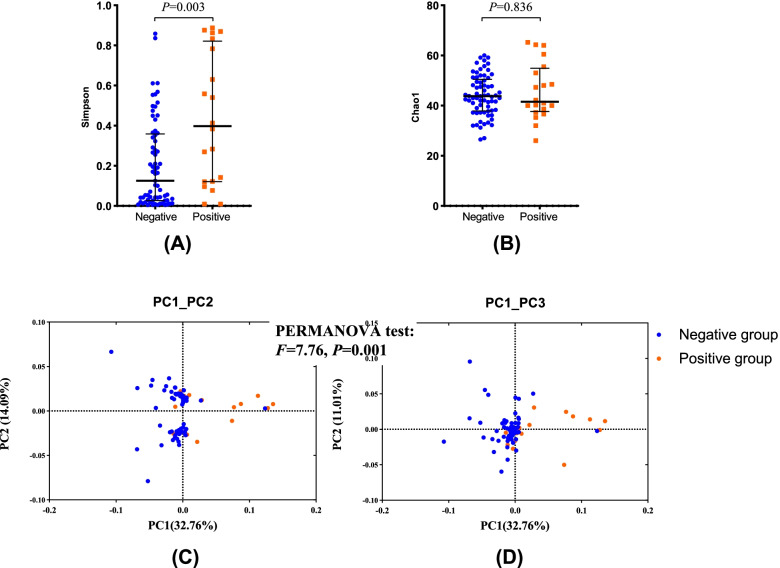
The α and β diversity between *M. hominis* positive and negative groups. **A** Simpson index between groups; **B** Chao1 index between groups. The middle line indicates the medians, the bars indicate the interquartile ranges, and all the *P* values were from nonparametric Kruskal–Wallis tests. (**C**) and (**D**) are PCoA plots based on binary Jaccard distances

In terms of α diversity indices, the Simpson index of the positive group was significantly lower than that of the negative group (*P* = 0.003, Fig. 1A), which means that the microbiome diversity appeared to increase with *M. hominis* positivity. However, the Chao1 indices were not significantly different between groups (*P* = 0.836, Fig. 1B). From the PCoA analysis, two groups related to bacterial infection were separate: the positive spots gathered on the left but negative spots gathered on the right (Fig. 1C and D), and the difference had statistical significance (PERMANOVA test, R^2^ = 0.082, *P* < 0.001).

**Fig. 2:**
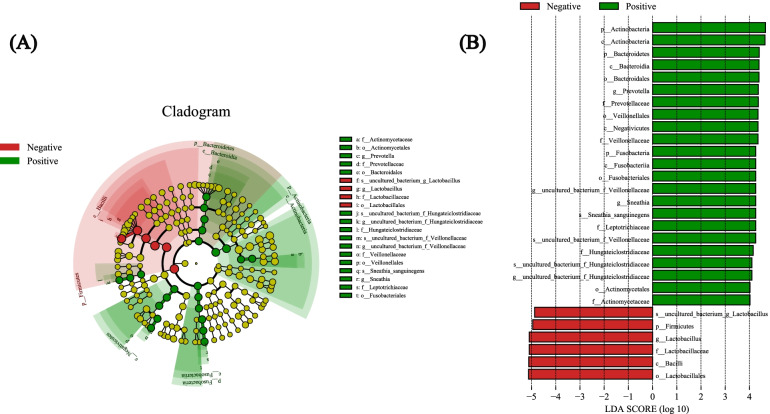
Lefse analysis based on *M. hominis* colonization status. Red stripe means the relative abundance was higher in *M. hominis* negative group, and green stripe means that it was higher in *M. hominis* positive group. Threshold of LDA score was 4.0

Further, we explored the potential *M. hominis*-related bacteria using Lefse analysis. Figure 2 shows that the *M. hominis* positive group had higher abundance of *Actinobacteria* class, *Bacteroidales* order, *Prevotella* genus, etc. Meanwhile, the *M. hominis* negative group had higher abundance of the *Lactobacillus* genus. At the species level, we explored the association between the relative abundance of *M. hominis* and the other main species. Table 2 shows that *M. hominis* was negatively correlated with *L. crispatus* (rho = − 0.24, *P* = 0.024) and positively correlated with *Gardnerella vaginalis*, *Atopobium vaginae* and *Prevotella bivia* (*P* all < 0.05), but it was not associated with *Ureaplasma parvum*.

**Table 2:** The Spearman correlation coefficients between *M. hominis* and other major species in the vaginal microbiome

Species	*rho*	*P*
*Lactobacillus crispatus*	−0.240	**0.024**
*Lactobacillus iners*	−0.017	0.875
*Lactobacillus gasseri*	−0.003	0.977
*Gardnerella vaginalis*	0.245	**0.021**
*Atopobium vaginae*	0.382	**0.001**
*Streptococcus*	−0.143	0.182
*Prevotella bivia*	0.215	**0.043**
*Ureaplasma parvum*	0.040	0.712

### Female fecundability and vaginal *M. hominis* colonization status

**Fig. 3:**
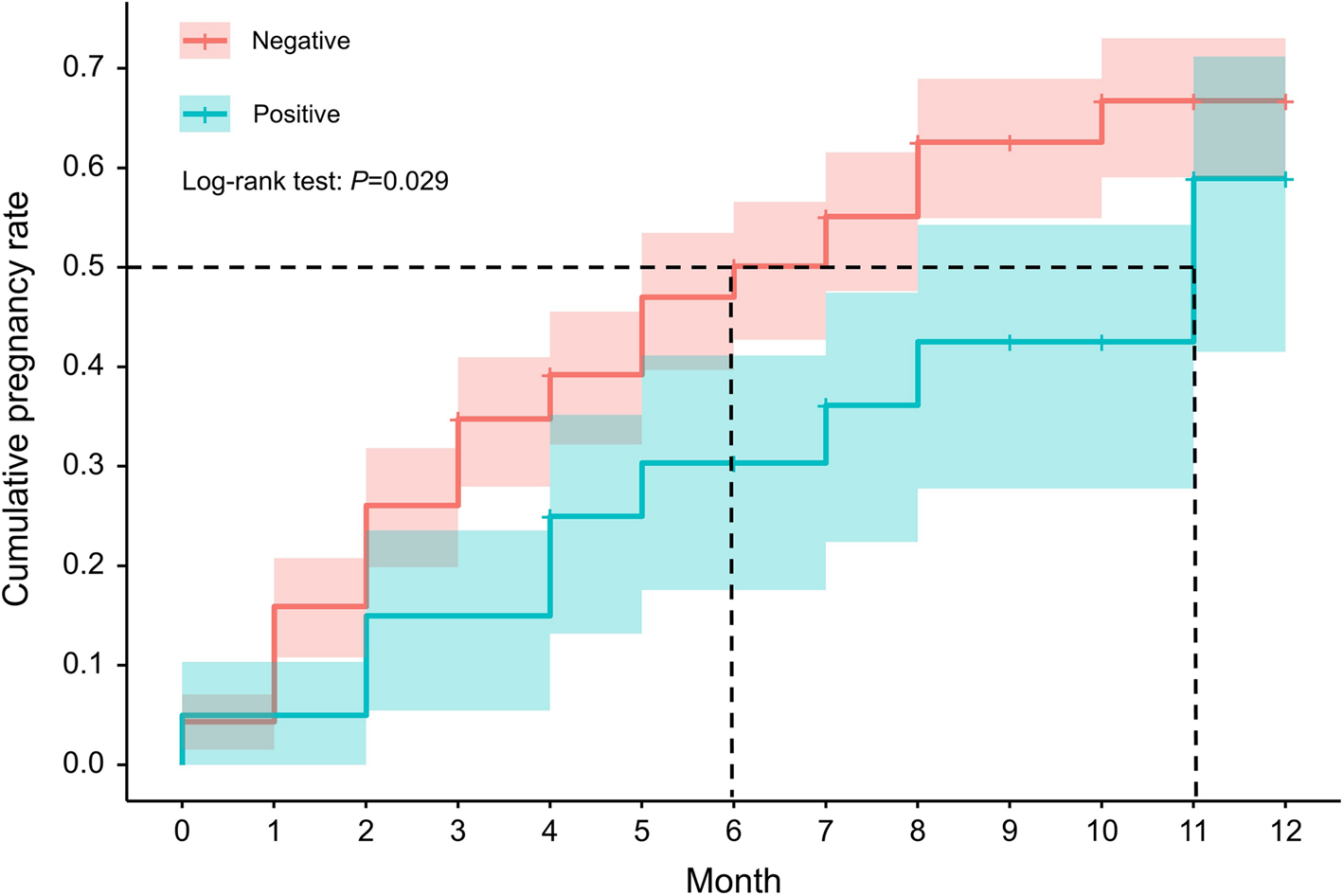
Kaplan–Meier plot for cumulative pregnancy rate based on *M. hominis* status

Overall, 51 women achieved pregnancy in 1 year. The median TTP for the *M. hominis* positive group was 6 months; that in the negative group was 11 months. The Kaplan–Meier method showed that the cumulative pregnancy rate for the *M. hominis* positive group was lower than that of the negative group (58.96% vs 66.76%, *P* = 0.029, Fig. 3).

After controlling for potential confounders, the risk of pregnancy in the *M. hominis* positive group was reduced by 38% compared with the negative group (FOR = 0.62, 95% CI: 0.42–0.93). After adjusting for the indices reflecting the vaginal microenvironment, including vaginal cleanliness grading and Shannon and Simpson index, this association remained (FOR = 0.63, 95% CI: 0.40–0.99, Table 3). This means that the *M. hominis* status has significance when evaluating female fecundability beyond the aggregative indicator of the vaginal microenvironment.

**Table 3:** The fecundability odds ratios for women with positive *M. hominis* status

*M.hominis* status	Crude FOR (95%CI)	Model A FOR (95%CI)	Model B FOR (95%CI)
Negative	Ref	Ref	Ref
Positive	0.65 (0.43-0.96)	0.62 (0.42-0.93)	0.63 (0.40-0.99)

Model A was adjusted by female age, pregnancy history, menstruation regularity and couple’s age difference;

Model B was additionally adjusted by vaginal cleanliness grading and Shannon and Simpson index based on Model A

## Discussion

In this prospective cohort, the pre-pregnant women with vaginal *M. hominis* colonization with low abundance had a unique vaginal microbiota structure, and had 37% lower fecundability. This association was independent from the overall vaginal microenvironment. This finding suggests the importance of *M. hominis* screening for women planning pregnancy.

Many epidemiological studies have found that vaginal *M. hominis* is an opportunistic pathogen. The prevalence of *M. hominis* has ranged between 3.1 and 15% in non-pregnant sexually active symptomatic and asymptomatic women [8]. The bacterial load of *M. hominis* increases in the dysbiosis of BV,[9, 16] but one third of women with BV do not carry *M. hominis*; therefore, it is not a suitable biomarker for BV diagnosis [17, 18]. Miyoshi et al. [10] found that a positive vaginal *M. hominis* culture is an independent predictive factor for preterm birth in patients with symptomatic threatened preterm labor. *M. hominis* infection was also associated with postpartum endometritis among women undergoing a cesarean section [19]. The association between *M. hominis* and female infertility is controversial. A meta-analysis showed that, in Iran, infertile women had a high prevalence of *M. hominis *[20]. Ma et al. [12] reported a similar finding that *M. hominis* is a potential risk factor for female infertility, but the effect size was small (OR = 1.56, 95% CI: 1.02–2.38). Previous studies have always used a case–control design, which leads to difficulty in making a causal inference because we cannot infer whether the *M. hominis* infection was earlier than the infertility. Thus, our study provides more convincing evidence that *M. hominis* infection reduces female fecundability, by using a prospective design.

The development of genomics has increased the understanding of the pathogenic mechanisms of *M. hominis*. In sequenced amniotic fluid/placental isolates, Allen [21] identified two new genes from *M. honimis* that encode surface-located membrane proteins, which is associated with colonization and/or infection of the upper reproductive tract during pregnancy and with preterm birth. Additionally, a surface lipoprotein, MHO_0730, has been found to have effects on promotion of infection and modulation and evasion of innate immunity [22]. Previous studies have suggested that vaginal pathogenic bacteria lead to ascending infection and fallopian tube adhesion,thus causing tubal infertility [23]. *M. hominis* can also cause direct tubal damage or altered ciliary activity within the fallopian tubes [24]. However, many unexplained cases of infertility could also be attributed to the vaginal microbiome. Researchers have put forward many hypothesis to explain it, including the impact of vaginal bacteria on sperm motility, [25] bioactive metabolites of the vaginal microbiome entering the blood to cause fecundability reduction, [26] and the potential effect of the vaginal microbiome on the hypothalamic pituitary ovarian axis [27]. Although there is no direct evidence that *M.hominis* would impact female fertility through above three pathways, these hypotheses are also reasonable for *M.hominis*. Additionally, *M.hominis* had been found to be associated with local inflammatory level, which might participate in the regulation of female fertility [28]. Further studies are needed to explore the specific mechanisms that explain the association between vaginal *M. hominis* colonization and female TTP.

This study is strengthened by its prospective design, and the TTP was used to evaluate fecundability. According to clinical practice guidelines, infertility should be diagnosed when a women has not become pregnant after having 12 months of regular, unprotected intercourse [29]. This means that women with TTP of more than 12 months are more likely to have infertility, so TTP is an indicator with clinical significance. In previous case–control studies, it was hard to investigate the vaginal microbiome before the definitive diagnosis, but our study overcame this problem. In addition, many sensitivity analysis were used to demonstrate the robustness of our results. Our study suggested that, whatever the status of the vaginal microbiome, detection of *M. hominis* is meaningful for evaluation of fecundability, because this association is independent from the vaginal microenvironment.

There are several inevitable limitations to this study. First, although the high-throughput sequencing method we used is a frequently used method of bacterial detection, it is not yet popular in clinics. Some samples with low abundance of *M. hominis* would not be accurately identified by culture. Therefore, our results lack comparability with other studies. Second, some researchers think that the vaginal microbiome shows dynamic change within one cycle, so our one-time assessment may not reflect the vaginal microenvironment in its entirety. Third, like all other prospective TTP related studies, there is inevitably selection bias [30]. Because the couples who have difficulties regarding pregnancy would be more likely to participate this program, however, unintended pregnant women could not be included in our study. Fourth, our strict exclusion criteria might increase selection bias, especially the women who were menstrual period when they took part in this program were excluded because *M. hominis* information could not be get. The future work should require the participants to collect vaginal swabs at a same fixed time in their menstrual cycle. Additionally, 13 women who refused to continue the follow-up without exact TTP in the first 3 months were excluded, which might expand selection bias. Fifth, our findings needs to be further validated because many potential confounding factors were not collected and did not be taken into account, such as male partners’ sperm quality, *M. hominis* infection status, and the couples’ regularity of sexual relations. In addition, couples’ educational levels is also important to be considered, because it can indirectly reflect the ability of couples to identify the fecundity period in the female menstrual cycle. Lastly, our findings should be interpreted cautiously, because all the participants were from Nanjing, China, and the sample size is small. The vaginal microenvironments ae diverse among females with different ethnicity, [31] and therefore whether this finding is generalizable to other pregnancy-planning women requires further study.

In summary, vaginal colonization by *M. hominis* in women pre-pregnancy is negatively correlated with female fecundability. *M. hominis* screening is necessary for pre-pregnancy health examination.

## Supplementary Information

**Additional file 1 Supplementary text**. The formula about Chao1 and Simpson indices

## Data Availability

The genome sequences can be accessed at https://www.ncbi.nlm. nih.gov/bioproject/browse using Bioproject PRJNA741092.
