# Supplementary material for: The associations between low abundance of Mycoplasma hominis and female fecundability: a pregnancy-planning cohort study
Source: BMC Microbiol. 2022 May 5;22:121. doi: 10.1186/s12866-022-02545-7 (PMC9069813; doi:10.1186/s12866-022-02545-7)
Supplement: Supplementary file 1 — Additional file 1 Supplementary text. The formula about Chao1 and Simpson indices [file 12866_2022_2545_MOESM1_ESM.docx]

**Supplementary text. The formula about Chao1 and Simpson indices.**

$$Chao1=\frac{N+n_{1}(n_{1}+1)}{2(n_{2}+1)}$$

The N is the amount of the observed operational taxonomic unit (OTU), $n_{1}$ is the amount of the OTU with just one representative sequence, $n_{2}$ is the amount of the OTU with two representative sequences. Thus, a higher Chao1 index means a more diversity for microbiome. Meanwhile, OTUs with low abundance are considered in this index. [1]

$$Simpson=1-\sum_{i=1}^{s} {P_{i}}^{2}$$

$P_{i}$ is the proportion of a specific OTU (i) among all OTUs. s is the number of the OTU type. This, a lower Simpson index means a higher diversity for microbiome. [2]

***Reference:***

[1]. Chao, A. (1984) Nonparametric Estimation of the Number of Classes in a Population. Scandinavian Journal of Statistics, 11, 265-270.

[2]. He F, Hu X S. Hubbell's fundamental biodiversity parameter and the Simpson diversity index[J]. Ecology Letters, 2005, 8(4): 386-390.
